# Exploratory Study on the Application of Graphene Platelet-Reinforced Composite to Wind Turbine Blade

**DOI:** 10.3390/polym16142002

**Published:** 2024-07-12

**Authors:** Hyeong Jin Kim, Jin-Rae Cho

**Affiliations:** 1 Department of Mechanical Engineering, University College London, London WC1E 7JE, UK; hj.kim.22@ucl.ac.uk; 2 Department of Naval Architecture and Ocean Engineering, Hongik University, Jochiwon, Sejong 30016, Republic of Korea

**Keywords:** wind turbine blades, graphene-platelet-reinforced composites, exploratory study, mechanical characteristics, finite element structural analysis

## Abstract

With the growth of the wind energy market and the increase in the size of wind turbines, the demand for advanced composite materials with high strength and low density for wind turbine blades has become imperative. Graphene platelets (GPLs) stand out as highly premising reinforcements due to their exceptional physical properties, resulting in their widespread adoption in the composite industry in recent years. The present study aims to analyze the applicability of a graphene-platelet-reinforced composite (GPLRC) to wind turbine blades in terms of structural performance. A finite element blade model is constructed by referring to the National Renewable Energy Laboratory (NREL) 5 MW wind turbine, and its reliability is verified through a convergence test. The performance of the wind turbine blade is quantitatively examined in terms of the deflection and stress, natural frequencies, and twist angle. The applicability of the GPL-reinforced wind blade is explored through a comparison with wind blades manufactured with glass fiber and carbon nanotubes (CNTs). The comparison indicates that the performance of a wind blade can be remarkably improved by reinforcing with GPLs instead of traditional fillers, and the weight of not only the wind blade itself but also the wind turbine system can be remarkably reduced. The present results can be useful in the development of next-generation high-strength lightweight wind turbine blades.

## 1. Introduction

Concerns over fossil fuel depletion are gradually subsiding owing to the discovery of new oil and gas reserves and the development of new technologies, such as in the shale revolution; however, the negative effects caused by the use of fossil fuels, including global climate change, have been noticeably increasing. Thus, there is still high global interest in developing and securing eco-friendly and sustainable renewable energy resources to replace conventional fossil fuels. Among the renewable energy resources, wind energy has particularly high energy efficiency and low environmental damage, and it has been evaluated as the fastest-growing energy source in terms of global power generation along with solar energy. According to a report from IRENA [1], wind energy is expected to represent more than 30% of global electricity generation by 2050.

Along with the growth of the wind energy industry, research has been actively conducted on the economic efficiency and structural safety of wind power equipment. In particular, blades have a direct impact on the economic efficiency of wind power equipment, including through the power generation efficiency and installation cost, and the use of high-strength lightweight materials has been emphasized to improve them. In recent years, composites that apply glass or carbon fibers to the epoxy matrix have been mainly used for wind blades [2]. Several studies have been conducted to investigate the applicability of new materials to wind blades by analyzing their mechanical characteristics and economic efficiency. For example, Mengal et al. [3] compared the material properties of basalt fibers with those of glass and carbon fibers that were used for wind blades. They insisted that basalt fibers can partially replace the two existing glass and carbon fibers and improve economic efficiency. Chikhradze et al. [4] analyzed the mechanical properties of hybrid composites containing basalt, glass, and carbon fibers and predicted that basalt fibers could partially (20–30%) replace expensive carbon fibers. Ong and Tsai [5] investigated economic efficiency according to the proportion of carbon fibers in hybrid composites. For example, when 70 and 30% were applied as the proportions of carbon fibers, they predicted that the weight would decrease to 70 and 49% of the existing weight, and the manufacturing cost would increase by approximately 127 and 89%, respectively. Several studies that analyzed the applicability of natural fibers have also been published. Holmes et al. [6,7] applied bamboo epoxy laminated composites to wind blades and proved that they have sufficient strength and stiffness to replace conventional glass fiber composites through comparisons. Shen-xue et al. [8] experimentally evaluated the strength of wind blades comprising bamboo materials and proved that the bamboo materials have sufficient strength through a comparison with glass fibers. In addition, various research cases that applied new materials have been systematically organized in existing literature review papers [9,10,11].

Recently, the applicability of carbon-based nanomaterials with excellent material properties, such as carbon nanotubes (CNTs), as well as conventional composites, has been examined to improve the mechanical performance of various structures. In the case of CNT-reinforced composites (CNTRCs), however, their limited practical applicability has been pointed out due to their high production cost, anisotropic material properties, and uneven distribution [12,13,14,15]. Conversely, graphene-platelet-reinforced composites (GPLRCs) have relatively a low production cost and isotropic material properties, as well as superior mechanical properties to those of CNTRCs, and various cases that improved mechanical characteristics by applying GPLRCs can be found in the literature. As a representative case, Rafiee et al. [16] compared the mechanical properties of epoxy composites reinforced with GPLs and CNTs at weight fractions of 0.1% and showed that GPLRCs are superior to CNTRCs in terms of various mechanical properties, such as stiffness, strength, and fracture toughness. Following the preceding study, Rafiee et al. [17] experimentally evaluated the buckling strength of epoxy composite beams reinforced with GPLs and CNTs at a weight fraction of 0.1%. They found that GPL reinforcement increases the critical buckling strength by 51.5, 42.8, and 31.8% compared to epoxy without reinforcement, multi-walled CNT reinforcement, and single-walled CNT reinforcement, respectively. Parashar and Mertiny [18] evaluated the buckling strength of a plate reinforced with GPLs through finite element analysis and reported that the buckling strength of the plate reinforced with GPLs at a volume fraction of 6% is increased by 26% compared to that of the plate without reinforcement. This implies that structural failure due to buckling could be reduced by 26% in real-world applications in wind turbine blades. Feng et al. [19] reported that an epoxy beam reinforced with GPLs exhibits a significant reduction in deflection compared to a beam without reinforcement and that bending performance is most effectively improved when the upper and lower parts of the beam are reinforced with GPLs. Gholami and Ansari [20] conducted a large deflection analysis for a square plate reinforced with GPLs using the functionally graded distribution as a variable, and they confirmed that an increase in GPLs is directly related to an increase in the stiffness of the plate. They also found that the increased stiffness of the plate effectively reduces its maximum deflection. Bahaadini and Saidi [21] analyzed the aeroelastic behavior of FG multilayer GPLRC rotating blades under supersonic flow using the first-order shear deformation theory and extended Hamilton principle. They found that the natural frequency is significantly affected by the added amount and the distribution pattern of GPLs. Domnica et al. [22] presented the structural optimization of a composite for wind turbine blades using finite element analysis. They found that an increase in the number of layers in risk areas and their reduction toward the blade tip lead to increased resistance to static and dynamic loads. The orientation of layers can improve the static and dynamic behaviors. Ansal Muhammed et al. [23] numerically analyzed the behaviors of nano SiO_2_ and Al_2_O_3_ dispersed in E-glass fiber/epoxy composites under different loading conditions. Their numerical study pointed out that the nanocomposite with 1% Al_2_O_3_ performed well under edge loading and flap loading. Abu-Okail et al. [24] examined the effects of the dispersion of metallic and grapheme nanoparticles on micro-structural and mechanical characteristics of hybrid carbon/glass fibers reinforced with a polymer composite. They presented a successful method to synthesize glass, carbon, and hybrid FRPs by mixing nanocomposites using a high-frequency sonication technique for wind turbines. As such, some studies improved performance by applying GPLRCs to various structures; however, there is still no reported case in which GPLRCs have been applied to wind turbine blades. Due to the nature of wind blades, high-strength lightweight materials are required to increase power generation efficiency and reduce construction costs. As the size of wind turbines increases, the importance of the materials used in blades has been gradually increasing.

In this context, changes in the mechanical characteristics of wind blades with the application of GPLRCs are closely examined, and the applicability of GPLRCs as future materials is explored in this study. The elaborate finite element model (FEM) in this study was generated by referring to the National Renewable Energy Laboratory (NREL) 5 MW offshore wind turbine blades. Through a numerical analysis that utilizes the finite element method, various mechanical characteristics (e.g., deflection, twist, stress, and natural frequency), which are the main considerations in wind blade design, are parametrically examined according to the volume fraction, functionally graded (FG) distribution pattern, and reinforcement location of GPLs. The mechanical characteristics of wind blades reinforced with GPLs, which are obtained through in-depth parametric numerical analysis, are investigated through a comparison with conventional glass fiber composites and CNTRCs to examine the applicability and superiority of GPLRCs as next-generation wind blade materials.

## 2. Materials and Finite Element Models

### 2.1. Material Modeling of Nanocomposites

In this study, we intend to analyze the mechanical characteristics of wind blades to which nanocomposites, such as GPLRCs and CNTRCs, as well as conventional glass fiber composites, are applied. It is practically difficult, however, to numerically implement the detailed models in nanometers in finite element analysis, and the accuracy of finite element analysis significantly decreases if the length scale of nanomaterials increases, as can be observed in the study by Kim and Cho [25]. Therefore, nanocomposites are homogenized in this study using the effective material properties calculated in terms of the material properties of the nanomaterials and the matrix. Polymethyl methacrylate (PMMA) is selected as the matrix, and (10,10) single-walled carbon nanotubes (SWCNTs) are taken as the CNTs. Table 1 represents their material properties by referring to Rafiee [17] and Shen and Zhang [26]. Here, E is the elastic modulus, G is the shear modulus, and ν is Poisson’s ratio. Subscripts 1 and 2 represent coordinates x and z in the blade span and thickness directions, respectively. Based on the values presented in Table 1, the effective elastic modulus $E_{eff}$ is evaluated using the Halpin–Tsai equations for GPLRCs and the modified rule of mixture for CNTRCs, as addressed in Appendix A. Meanwhile, the effective Poisson ratio $\nu_{eff}$ and density $\rho_{eff}$ are calculated according to the simplest linear rule of mixtures [27] as follows.

(1) $$\nu_{eff}=V_{n}\;\nu_{n}+V_{m}\;\nu_{m}$$

(2) $$\rho_{eff}=V_{n}\;\rho_{n}+V_{m}\;\rho_{m}$$

Here, $V_{n}$ and $V_{m}$ are the volume fractions of the nanomaterial and matrix. Subscripts *eff*, *n*, and *m* denote the effective material property, nanomaterial (GPL or CNT in this study), and matrix, respectively.

**Figure 1:**
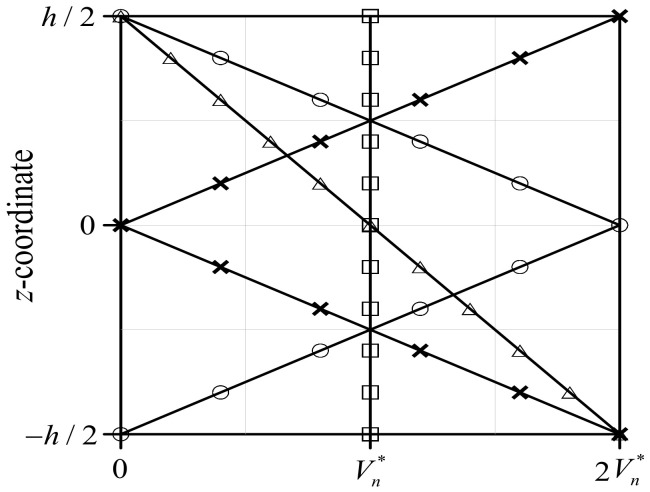
Thickness-wise volume fraction distributions $V_{n}$ in functionally graded nanocomposites (□: FG-U, △: FG- Λ, ○: FG-O, **×**: FG-X).

Because the behavior of a structure containing nanofillers may significantly vary depending on the volume fraction and functionally graded distribution of the nanofillers, these need to be considered when changes in the mechanical characteristics of the wind blade are examined. Therefore, four functionally graded distribution patterns of GPLRCs, that is, FG-U, FG-Λ, FG-O, and FG-X, are considered in this study, as shown in Figure 1.

Here, the volume fractions $V_{n}\left(z\right)$ of nanofillers for each distribution pattern in the thickness direction are expressed as

(3) $$V_{n}(z)=\left\{\begin{array}{cc} V_{n}^{*}, & \text{FG-U}\\ (1-2z/h)V_{n}^{*}, & \text{FG-Λ}\\ 2(1-2\left|z\right|/h)V_{n}^{*}, & \text{FG-O}\\ 2(2\left|z\right|/h)V_{n}^{*} & \text{FG-X} \end{array}\right.$$

where h is the structure’s thickness, and z is the coordinate in the thickness direction. In addition, the total volume fraction $V_{n}^{*}$ of the nanofillers is calculated as

(4) $$V_{n}^{*}=\frac{m_{n}}{m_{n}+\rho_{n}(1-m_{n})/\rho_{m}}$$

where $\rho_{n}$ and $m_{n}$ are the density and mass fraction of the nanofillers, respectively, and $\rho_{m}$ is the matrix density.

### 2.2. Finite Element Model

**Figure 2:**
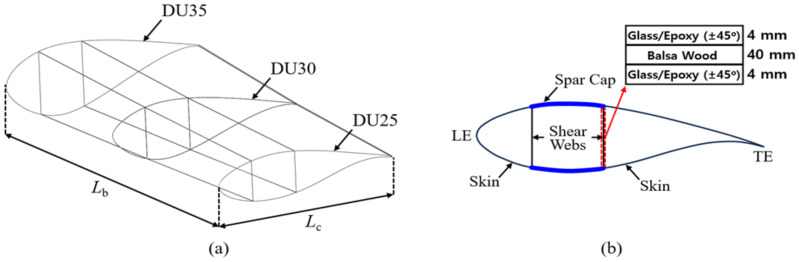
Partial model of the wind turbine blade: (**a**) overall view; (**b**) cross-sectional view.

The finite element method has been widely used to predict the static and dynamic behaviors of wind turbine blades manufactured with various composites [28]. In this study, a partial model of a wind blade is generated using the airfoils utilized in the NREL 5 MW wind turbine model, as shown in Figure 2a [29]. As shown in the model, the length of the blade $L_{b}$ is 8.2 m, and the chord lengths of the three cross-sections are set by 4.007 m (DU25), 4.249 m (DU30), and 4.458 m (DU35), respectively. The blade skin is idealized into a composite layer (skin) rather than modeling the leading edge (LE), LE panel, trailing edge (TE), and TE panel of the blade, as shown in Figure 2b. In this instance, the thickness of the blade skin t is set to 40 mm in reference to Cox and Echtermeyer [30]. The shear web is modeled as a sandwich panel, which consists of a 40 mm thick balsa wood core and two facesheets. Each facesheet is composed of four layers: a 1 mm thick glass fiber/epoxy layer with (+45°/−45°/+45°/−45°) orientation axis angles. Table 2 shows the material properties of the balsa wood and glass fiber/epoxy composite used in the model [30,31].

The finite element model is created using midas-NFX 2024R1 [32], a commercial FEM software program. The FEM mesh of the model is created using composite material shell elements that calculate the internal force and stiffness based on the composite material lamination theory; the thickness-wise functionally graded distribution of material properties is implemented by generating a number of homogeneous layers with different isotropic material properties in the thickness direction. To determine the optimal finite element size, a convergence study is conducted in terms of the flap-wise deflection w and twist angle θ of the blade model.

**Figure 3:**
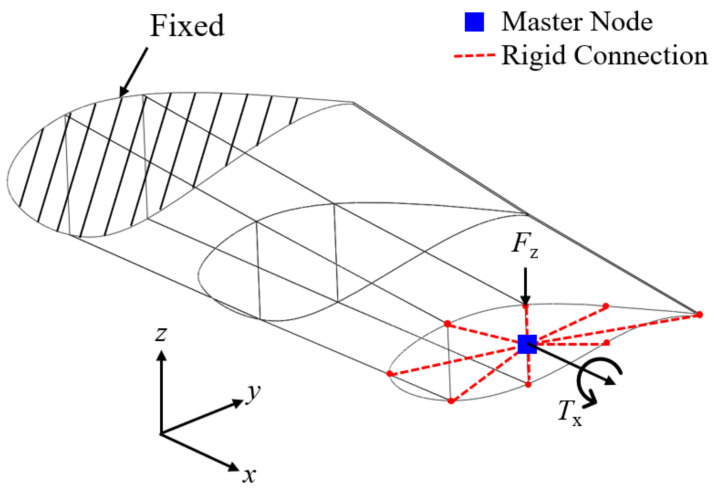
Boundary and loading conditions of the finite element wind blade model.

**Figure 4:**
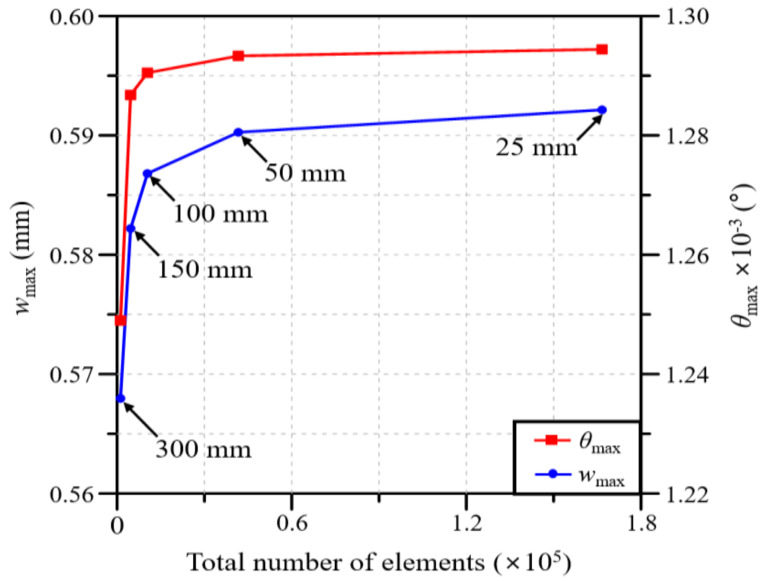
Preliminary convergence study for determining the critical element size for the blade’s finite element mesh.

Figure 3 shows the boundary and loading conditions of the partial blade model set in this study. The deflection and twist are computed under a concentrated load $F_{z}=10\;\mathrm{kN}$ and torsional load $T_{x}=10\;\mathrm{kN}\cdot m$, respectively. Each load is applied to the master node connected to the nodes at the blade tip through the rigid link elements, as shown in Figure 3. Figure 4 represents the variations in the deflection w and twist angle θ with respect to the total number of elements. The numbers in the figure represent the element size. Based on the results of the convergence study, the optimal element size of the FE model is chosen to be 50 mm, which corresponds to 41,656 elements in total. When compared with a smaller mesh size (i.e., 25 mm), the chosen mesh size provides a shorter CPU time by 6.2 times even though the numerical quality is deteriorated with relative errors of 0.3% in $w_{\max}$ and 0.1% in $\theta_{\max}$.

## 3. Results and Discussion

### 3.1. Material Type

**Figure 5:**
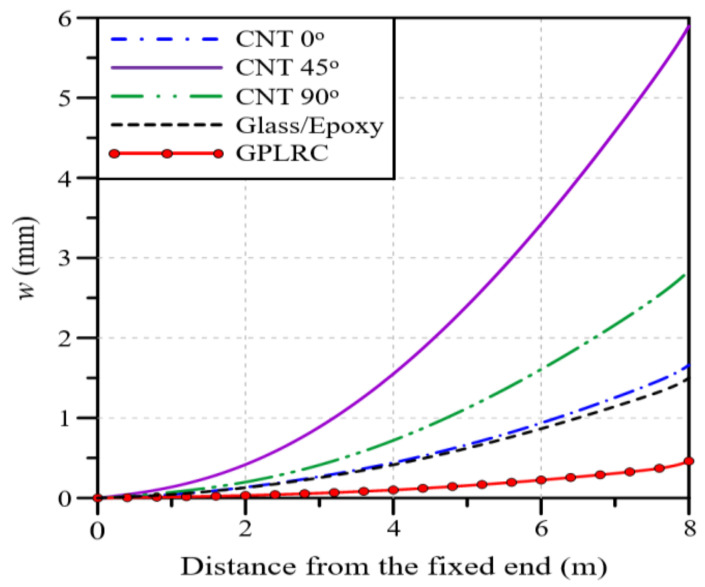
Variations in the deflection of the partial blade model along the blade span for different materials.

In this section, the mechanical characteristics of a wind blade reinforced with GPLs are analyzed under various analysis conditions, and the results are compared with those of conventional glass/epoxy composites and a CNTRC. In the case of the CNTRC, the mechanical properties of the composite significantly vary depending on the orientation angle of the principal axis of the CNTs because it has anisotropic material properties. Therefore, three orientation angles of the principal axis of the CNTs (0°, 45°, and 90°) are considered in this study. In addition, the total volume fractions of GPLs and CNTs ($V_{GPL}^{*}$ and $V_{CNT}^{*}$) are fixed at 0.12 to set identical analysis conditions. Figure 5 represents the blade deflection along the blade span for each material. It is observed that CNT 45° produces the largest deflection, followed by CNT 90°, CNT 0°, glass/epoxy, and the GPLRC. When the GPLRC is used, the maximum deflection is reduced by 69% compared to the glass/epoxy and by 92% compared to CNT 45°. It should be noted that the matrix is commonly PMMA for CNTRCs and GPLRCs.

**Figure 6:**
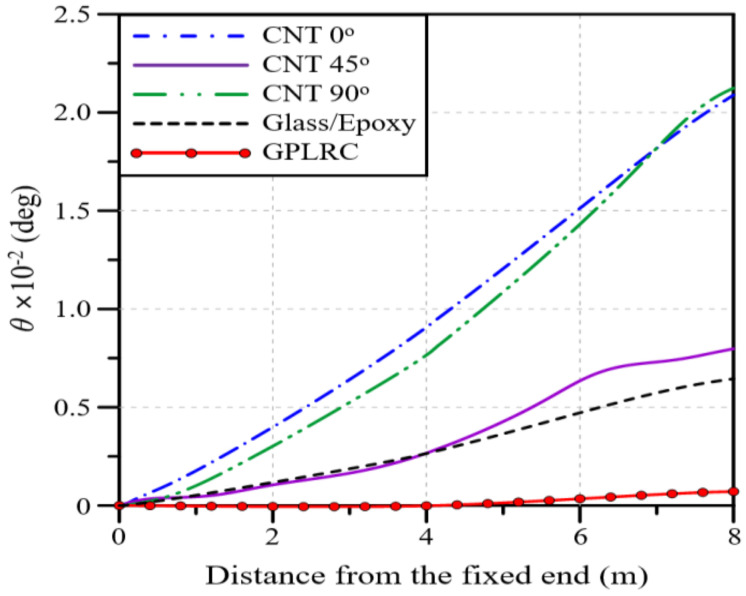
Twist angles of the partial blade model along the blade span for different materials.

Figure 6 presents the twist angle of the blade model along the blade span for each material. CNT 90° produces the largest maximum twist angle, followed by CNT 0°, CNT 45°, glass/epoxy, and the GPLRC, which shows a tendency slightly different from that of deflection. When the GPLRC is adopted, the maximum twist angle is reduced by 89% compared to glass/epoxy and by 97% compared to CNT 90°. Thus, it is found that the GPLRC exhibits the smallest deflection and twist angle, followed by glass/epoxy and CNTRC.

Next, a modal analysis was conducted to examine the differences in the natural frequency of the partial blade model depending on the material type. Because a partial blade model is used in this study, there may be differences in the analysis results compared to those of a full blade model. The results, however, are judged to be valid in identifying the relative change in natural frequency depending on the material type. Table 3 shows a comparison of the natural frequencies of three low-order modes for each material. A close examination of the analysis results reveals that the natural frequencies are higher in the order of the GPLRC, glass/epoxy, and CNTRC. In the case of the CNTRC, the results are different depending on the axis orientation angle and mode order. Here, note that the GPLRC exhibits much higher natural frequencies than the other materials. The three types of results indicate that the GPLRC has the highest performance in terms of lateral stiffness, torsional stiffness, and relative vibratory stiffness with respect to the blade mass, followed by glass/epoxy and the CNTRC, which is slightly affected by the orientation of the principal axis of the CNTs.

### 3.2. Functionally Graded Distribution

A functionally graded material (FGM) is a stacked composite in which the volume fractions of the constituent particles change continuously and functionally from one side to the other in a specific direction. Because FGMs are effective in relieving structural defects that occur in traditional lamination-type composites due to the sharp discontinuity in the material property distribution (e.g., stress concentration, plastic deformation, and delamination), various research cases have adopted FGMs. As introduced in the literature reviews by Zhao et al. [33] and Liew et al. [34], studies have been actively conducted to analyze changes in the mechanical properties of nanocomposites, such as GPLRCs and CNTRCs, depending on the functionally graded distribution of material volume fractions.

**Figure 7:**
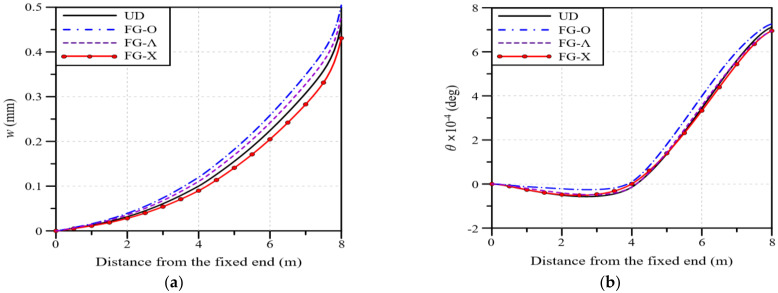
Effects of the thickness-wise GPL distribution pattern on the variation along the blade span: (**a**) deflection; (**b**) twist angle.

In this context, the impact of the functionally graded distribution pattern of GPLs on the deflection and twist angle of the wind blade is examined. The functionally graded distribution of GPLs is numerically implemented by calculating the effective material properties in the thickness direction using the homogenization equations introduced in Section 2 and Appendix A. Here, the total volume fraction of GPLs, $V_{GPL}^{*}$, is fixed at 0.12 regardless of the GPL distribution pattern. Figure 7 presents the deflection and twist angle of the wind blade for the four different functionally graded distribution patterns of GPLs. It can be observed that FG-O exhibited the largest deflection and twist angle, followed by FG-Λ, FG-U, and FG-X. Thus, FG-X produces the highest structural stiffness because GPLs with a larger elastic modulus are biased towards the top and bottom, where bending and torsional shear stresses are largest. On the other hand, the total weights of the wind blades are the same for the four FG distribution patterns because the total GPL volume fractions are kept the same. Compared to the results of comparing the deflection and twist angle according to the material type (Figure 5 and Figure 6), however, the difference depending on the functionally graded distribution pattern is quite small. Nonetheless, using the FG-X distribution pattern to improve the deflection and twist performance of a blade reinforced with a GPLRC is preferable, even though it is not advisable to expect a significant improvement, as the performance difference depends on the material type.

### 3.3. Volume Fraction

**Figure 8:**
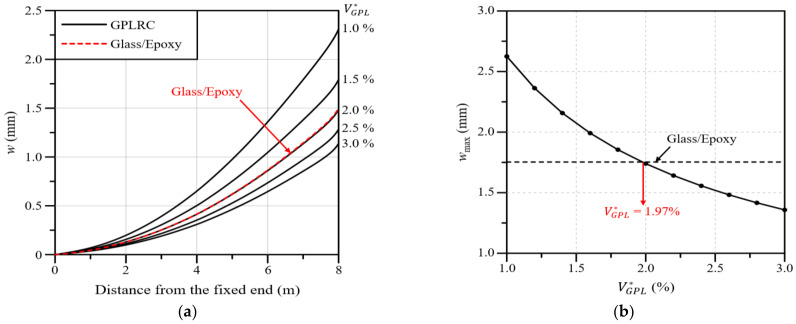
Variations: (**a**) Deflection along the blade span for different volume fractions; (**b**) the maximum deflection with respect to the GPL volume fraction.

In this section, the mechanical properties of the wind blade according to the total volume fraction of GPLs, $V_{GPL}^{*}$, are analyzed. In addition, through a comparison with the glass/epoxy composites used in wind blades, a $V_{GPL}^{*}$ value that exhibits similar performance is estimated. The total volume fraction of GPLs, $V_{GPL}^{*}$, is presented as a percentage rather than a decimal fraction so that it can be intuitively understood. Figure 8a shows the blade deflection along the blade span for different $V_{GPL}^{*}$ values. The analysis results show that the deflection decreases as the $V_{GPL}^{*}$ value increases, and the deflection is similar to that of the glass/epoxy composites at $V_{GPL}^{*}=2.0\%$. Figure 8b shows the maximum deflection according to the $V_{GPL}^{*}$ value. It can be observed that the maximum deflection is identical to that of the glass/epoxy composites at $V_{GPL}^{*}=1.97\%$.

**Figure 9:**
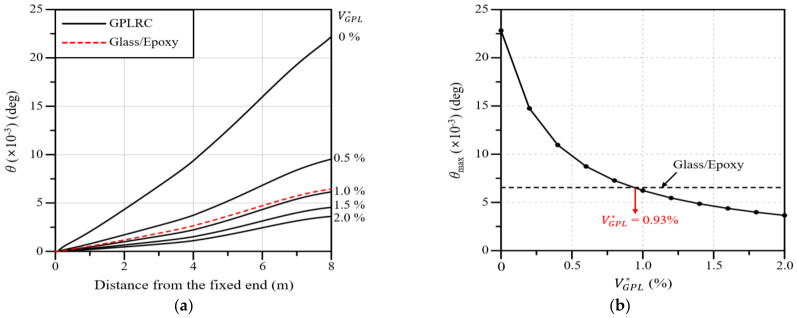
Variations: (**a**) Twist angle along the blade span for different volume fractions; (**b**) the maximum twist angle with respect to the GPL volume fraction.

**Figure 10:**
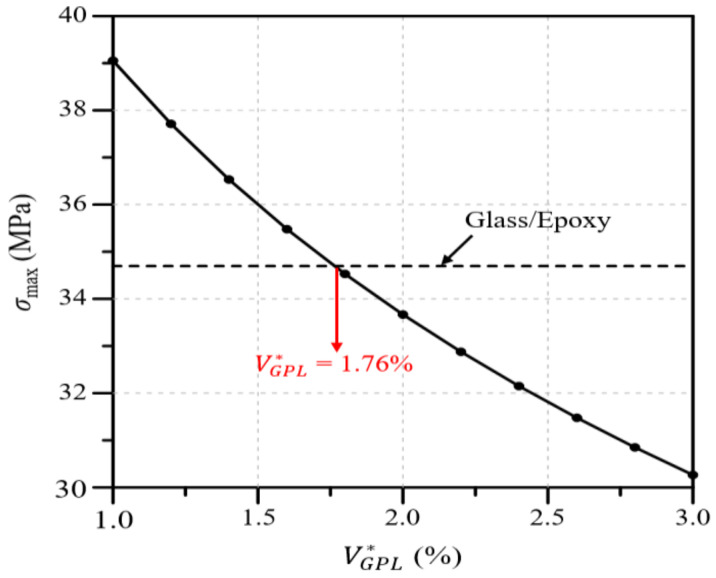
Variations in the maximum von Mises stress with respect to the GPL volume fraction.

Figure 9a presents the twist angle of the wind blade along the blade span for different values of $V_{GPL}^{*}$. The analysis results show that the twist angle gradually decreases as the value of $V_{GPL}^{*}$ increases, and the twist angle is similar to that of the glass/epoxy composite at $V_{GPL}^{*}=1.0\%$. Moreover, it can be observed that the reduction in the twist angle gradually decreases and tends to converge as the value of $V_{GPL}^{*}$ increases. Figure 9b presents the maximum twist angle according to the value of $V_{GPL}^{*}$. Similarly to the results of Figure 10, the maximum twist angle is the same as that of the glass/epoxy composite at $V_{GPL}^{*}=0.93\%$, and the twist angle reduction gradually tends to converge as the value of $V_{GPL}^{*}$ increases.

Next, Figure 10 presents the maximum von Mises stress in the wind blade according to the value of $V_{GPL}^{*}$. As with the deflection and twist angle results, the maximum von Mises stress decreases as the value of $V_{GPL}^{*}$ increases, and the maximum stress is the same as that of the glass/epoxy composite at $V_{GPL}^{*}=1.76\%$. Summarizing all of the analysis results presented in this section, the deflection, twist angle, and stress of the wind blade decrease as the value of $V_{GPL}^{*}$ increases, and the same maximum values as those of the glass/epoxy composite are observed when the values of $V_{GPL}^{*}$ are 1.97, 0.93, and 1.75%, respectively.

### 3.4. GPL-Reinforced Blade Elements

**Figure 11:**
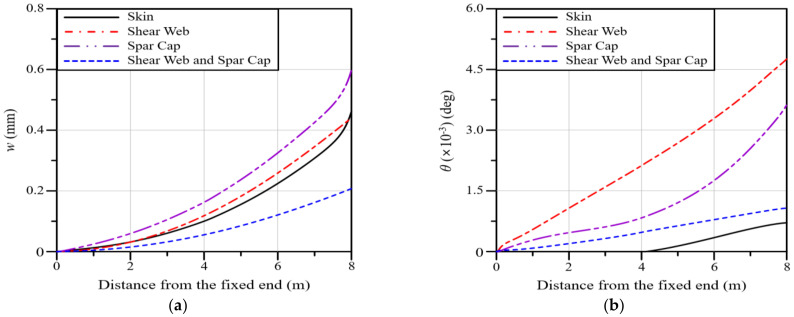
Variations along the blade span for different GPL−reinforced blade elements: (**a**) deflection; (**b**) twist angle.

In this section, the deflection and twist angle according to GPL-reinforced blade elements in a wind blade are analyzed. The GPL-reinforced elements are divided into the skin, shear web, and spar cap, as shown in Figure 2b. Four cases, including an additional case considering both the shear web and spar cap, are analyzed. As the total volume of GPLs varies depending on the GPL-reinforced blade element, the analysis was performed such that the same content of GPLs could be inserted regardless of the reinforced blade element. Figure 11a represents the deflection along the blade span for four different GPL-reinforced blade elements. The analysis results reveal that the spar cap shows the largest deflection, followed by the shear web, skin, and shear web and spar cap. In the case of the shear web and spar cap, the deflection is significantly smaller than that in the other three cases.

Figure 11b represents the twist angle along the blade span for four different GPL-reinforced blade elements in the GPLRC. The twist angle increases in the following order: the shear web, spar cap, shear web and spar cap, and skin. This result is significantly different from the result for deflection in Figure 11a. This could be because the bending stiffness and torsional stiffness of the wind blade do not show similar tendencies depending on the GPL-reinforced blade element. Reductions in the deflection and twist angle lead to longer fatigue life because the fatigue toughness becomes larger in proportion to the GPL-reinforced amount, even though the stress level might not be remarkably altered.

## 4. Conclusions

The applicability of GPLRCs to wind blades was explored through a numerical analysis, for which a finite element wind blade model was developed by referring to the NREL 5 MW wind turbine model. The reliability of the developed finite element wind blade model was secured with the help of a convergence test. The performance of a wind blade was quantitatively examined in terms of the deflection and stress, natural frequencies, and twist angle. The applicability of a GPL-reinforced wind blade was evaluated through a comparison with wind blades reinforced with glass fibers and carbon nanotubes (CNTs). From the comparative numerical experiments, which took place under various conditions, the following main observations are drawn:

- Under a bending load, CNT 45° produces the largest deflection, followed by CNT 90°, CNT 0°, glass/epoxy, and the GPLRC. Under a torsional load, CNT 90° shows the largest twist angle, followed by CNT 0°, CNT 45°, glass/epoxy, and the GPLRC. For a blade reinforced with GPLs, the maximum deflection is reduced by 69 to 92% and the maximum twist angle is reduced by 89 to 97% compared to that of other materials.
- As the total volume fraction of GPLs $V_{GPL}^{*}$ increases, the deflection, twist angle, and stress of the wind blade gradually decrease. The same maximum values as those of the glass/epoxy composites are observed when the values of $V_{GPL}^{*}$ are 1.97, 0.93, and 1.75%, respectively.
- Overall, the deflection and twist angle according to the functionally graded distribution of GPLs are found to increase in the following order: FG-O, FG-Λ, FG-U, and FG-X. The difference, however, is relatively insignificant compared to the difference caused by the material type and the GPL volume fraction.
- The deflection and twist angle of the wind blade significantly vary depending on the GPL-reinforced blade element of the GPLRC. The spar cap exhibited the largest deflection, followed by the shear web, skin, and shear web and spar cap, while the shear web produced the largest twist angle, followed by the spar cap, shear web and spar cap, and skin.
- The comparative results suggest that the mechanical properties of wind blades can be significantly improved by introducing GPLs. It is also expected that a GPLRC, an ultra-lightweight material, can significantly reduce the weight of wind blades and the total wind turbine manufacturing cost by reducing the weight of blade support structures.

Meanwhile, this study employed the widely used FEM, but advanced and comprehensive simulation techniques, such as the multi-scale modeling, are needed to more accurately predict the performance of these nanocomposites under various loading conditions by bridging the scale gap between the nano-scale properties of graphene and the macro-scale behavior of the matrix material. Furthermore, the effects of the interactions between different types of nanofillers on the overall mechanical properties of nanocomposites used for wind turbine blades would be a worthy topic for future work.

## Figures and Tables

**Table 1:** The base material properties of PMMA, GPL, and SWCNTs [17,26].

Material	$E_{11}$ (GPa)	$E_{22}$ (GPa)	$G_{12}$ (GPa)	$\nu_{12}$	ρ (kg/m^3^)
PMMA	2.5	2.5	0.9	0.340	1150
GPL	1010.0	1010.0	425.8	0.186	1060
SWCNT (10,10)	5646.6	7080.0	1944.5	0.175	1400

**Table 2:** The material properties of the balsa wood and glass fiber/epoxy composite [30,31].

Material	$E_{11}$ (GPa)	$E_{22}$ (Gpa)	$G_{12}$ (GPa)	$\nu_{12}$	ρ (kg/m^3^)
Balsa wood	0.4	0.4	0.16	0.1	125
Glass fiber/Epoxy	41.0	9.0	4.10	0.3	1890

**Table 3:** Comparison of the natural frequencies of the partial blade model for different materials.

Mode	Natural Frequencies (rad/s)				
	CNT 0°	CNT 45°	CNT 90°	Glass/Epoxy	GPLRC
Ⅰ	56.791	45.627	58.452	63.917	181.890
Ⅱ	74.952	102.238	74.794	84.925	282.161
Ⅲ	105.228	123.894	90.356	105.343	325.399

## Data Availability

The original contributions presented in this study are included in the article; further inquiries can be directed to the corresponding author.

## Appendix A. Effective Material Properties

GPLs are assumed to act as an effective rectangular solid, and graphene-reinforced composites are modeled as an isotropic material with effective material properties [35]. The effective elastic modulus $E_{eff}$ of GPLRCs is estimated using the Halpin–Tsai micromechanical model [36] as follows:

(A1) $$E_{eff}=\frac{3}{8}\cdot \frac{1+\xi_{L}\eta_{L}V_{GPL}}{1-\eta_{L}V_{GPL}}E_{m}+\frac{5}{8}\cdot \frac{1+\xi_{T}\eta_{T}V_{GPL}}{1-\eta_{T}V_{GPL}}E_{m}$$

(A2) $$\eta_{L}=\frac{E_{GPL}-E_{m}}{E_{GPL}+\xi_{L}E_{m}},\;\;\;\eta_{T}=\frac{E_{GPL}-E_{m}}{E_{GPL}+\xi_{T}E_{m}}$$

where the subscripts L, T, GPL, and m represent the longitudinal and transverse directions, graphene platelet, and matrix, respectively. $\xi_{L}$ and $\xi_{T}$ are geometric parameters given by

(A3) $$\xi_{L}=\frac{2l_{GPL}}{t_{GPL}},\;\;\xi_{T}=\frac{2w_{GPL}}{t_{GPL}}$$

with the length $l_{GPL}$, width $w_{GPL}$, and thickness $t_{GPL}$ of the GPLs. In this study, the geometric dimensions of the GPLs were chosen by referring to Rafiee et al. [15], where $l_{GPL}=2.5\;\mu m$, $w_{GPL}=1.5\;\mu m$, and $t_{GPL}=1.5\;\mu m$, respectively.

It is worth noting that the above assumption makes the Halphin–Tsai theory unrealistic when the GPL volume fraction is large. The anisotropy caused by the GPL orientation, packing arrangement, and spacing in the matrix/resin could be evaluated by employing the Tsai–Wu or checkerboard micromechanical models. The former was proposed in the context of generally anisotropic materials by using a quadratic polynomial expression of stresses with tensorial coefficients [37]. In the latter model, the GPL elements are assumed to be randomly dispersed in a checkerboard configuration such that some blocks denote GPL platelets, while the other blocks denote the resin matrix. The effective elastic modulus of the GPLRC is evaluated with an appropriate simulation technique [38].

Meanwhile, the effective elastic modulus $E_{eff}$ and shear modulus $G_{eff}$ of the CNTRC are estimated using the modified linear rule of mixtures such that

(A4) $$E_{11,eff}=\eta_{1}V_{CNT}(z)E_{11,CNT}+V_{m}E_{m}$$

(A5) $$\frac{\eta_{2}}{E_{22,eff}}=\frac{V_{CNT}(z)}{E_{22,CNT}}+\frac{V_{m}}{E_{m}}$$

(A6) $$\frac{\eta_{3}}{G_{12,eff}}=\frac{V_{CNT}(z)}{G_{12,CNT}}+\frac{V_{m}}{G_{m}}$$

where the subscripts 1 and 2 represent the coordinate axes x and z in the direction of the length and thickness of the CNTRC, and the subscripts CNT and m represent carbon nanotubes and the matrix, respectively. The CNT efficiency parameters $\eta_{j}$ (j = 1, 2, 3) are as follows: 0.137, 1.022, and 0.715 for $V_{CNT}^{*}=0.12$, 0.142, 1.626, and 1.138 for $V_{CNT}^{*}=0.17$, and 0.141, 1.585, and 1.109 for $V_{CNT}^{*}=0.28$.
